# Effects of a Video-Guided Active Break Programme on the Self-Esteem and Socio-Emotional Well-Being of Schoolchildren with Special Educational Needs: Active Classes Project

**DOI:** 10.3390/bs16030459

**Published:** 2026-03-19

**Authors:** Alejandra Robles-Campos, Yasna Chávez-Castillo, Isidora Zañartu, Ana María Arias, Carolina Muñoz, José Guzmán, Daniel Reyes-Molina, Igor Cigarroa, Maria Antonia Parra-Rizo, Juan de Dios Benítez-Sillero, Jose Manuel Armada-Crespo, Javier Murillo-Moraño, Rafael Zapata-Lamana

**Affiliations:** 1 Escuela de Educación, Universidad de Concepción, Los Ángeles 4451032, Chile; alejandrarobles@udec.cl (A.R.-C.); yasnasolchavez@udec.cl (Y.C.-C.); anamarias@udec.cl (A.M.A.); caromunozp@udec.cl (C.M.); josguzman@udec.cl (J.G.); 2 Prograna de Doctorado en Psicología, Universidad de Concepción, Concepción 4070386, Chile; izanartu@udec.cl; 3 Facultad de Salud, Escuela de Kinesiología, Universidad Santo Tomás, Los Ángeles 4440000, Chile; danielreyes@udec.cl (D.R.-M.); rafaelzapata@udec.cl (R.Z.-L.); 4 Escuela de Kinesiología, Facultad de Ciencias de la Salud, Universidad Católica Silva Henríquez, Santiago 8330225, Chile; icigarroac@ucsh.cl; 5 Departamento de Psicología de la Salud, Facultad de Ciencias Sociales y de la Salud, Campus de Elche, Universidad Miguel Hernández (UMH), 03202 Elche, Spain; maria.parrar@umh.es; 6 Facultad de Ciencias de la Salud, Universidad Internacional de Valencia (VIU), 46002 Valencia, Spain; 7 Grupo de Investigación en Deporte y Educación Física para el Desarrollo Personal y Social, Universidad de Córdoba, 14071 Cordoba, Spain; m62arcrj@uco.es (J.M.A.-C.); z02mumoj@uco.es (J.M.-M.); 8 Departamento de Didácticas Específicas, Universidad de Córdoba, 14071 Córdoba, Spain; 9 Centro de Magisterio “Sagrado Corazón”, Universidad de Córdoba, 14006 Córdoba, Spain; 10 Centro de Vida Saludable, Universidad de Concepción, Concepción 4070386, Chile

**Keywords:** active breaks, self-esteem, socio-emotional well-being, school, schoolchildren, special educational needs, randomised controlled trial

## Abstract

Serving students with special educational needs (SENs) involves recognising that their learning is closely linked to their emotional needs. Self-esteem and socio-emotional well-being play a key role in their motivation and adaptation to school. In this context, physical activity-based interventions at school emerge as a possible way to strengthen their self-esteem and socio-emotional well-being. The aim of this study was to analyse the effects of a web-based active break programme on self-esteem in students aged 6 to 10 years with SENs and on socio-emotional well-being in the subgroup of first–second-grade students. A pre-specified sub-analysis was conducted of a multicentre randomised controlled trial with a sample of 161 students with special educational needs (7.8 ± 1.1 years, 32% girls), divided into a control group (85 students) and an experimental group (76 students). A programme of video-guided active breaks was implemented in the classroom, applied twice a day, five days a week for 12 weeks, via a web platform. Self-esteem was assessed using the School Self-Esteem Test (SSET), and socio-emotional well-being was assessed using the Self-Report of Socio-Emotional Well-Being (SRSEWB). A significant Time × Group interaction was observed for self-esteem, F_(1, 157)_ = 5.43, *p* = 0.021, η^2^_p_ = 0.033, but no statistically significant effects were detected for socio-emotional well-being. These findings suggest that active break interventions may help strengthen self-esteem in students with SENs. Future research should examine the temporal stability of these improvements, determine the optimal intervention duration required to generate sustained changes, and evaluate longer-term socio-emotional outcomes.

## 1. Introduction

Students with special educational needs (SENs) are those who require additional, temporary, or permanent support to access the curriculum and participate fully in the school environment (UNESCO, 1995). Students with SENs often face academic, social, and emotional challenges that affect their adaptation to school, particularly their self-esteem, defined as the positive or negative assessment a person makes of themselves and their own abilities (Rosenberg, 1979) and their socio-emotional well-being, understood as the set of positive emotional experiences, adequate emotional regulation skills, and the perception of satisfactory interpersonal relationships (OECD, 2025).

Several studies have documented that students with SENs tend to have lower levels of self-esteem compared to their peers without SENs. For example, research has shown that students with intellectual disabilities tend to report more negative perceptions of themselves, especially in areas related to academic and social competence (Elbaum & Vaughn, 2001). Similarly, students on the autism spectrum show more fragile self-esteem linked to difficulties in social interaction and frequent experiences of stigmatisation (Hebron & Humphrey, 2014). This set of factors is also associated with lower socio-emotional well-being, as these experiences are linked to higher levels of anxiety, stress, and social isolation (Almeida et al., 2022). Thus, even in inclusive contexts, there is a persistent risk that these students will develop a less favourable self-concept and lower socio-emotional well-being if they do not receive adequate support and experiences of success at school (Cappadocia et al., 2012).

Although self-esteem and socio-emotional well-being are closely related, they represent conceptually distinct processes. Socio-emotional well-being encompasses broader dimensions such as emotional regulation, relational functioning, and sustained perceptions of belonging, which typically develop over longer periods and through more stable interpersonal interactions (Denham et al., 2012; S. M. Jones & Bouffard, 2012). In the school context, higher levels of socio-emotional well-being have been consistently associated with better classroom adjustment, stronger peer relationships, greater academic engagement, and lower levels of anxiety and behavioural difficulties (Durlak et al., 2011; A. S. Jones et al., 2015). Students who experience positive socio-emotional functioning are more likely to participate actively in class, persevere in the face of academic challenges, and establish supportive relationships with teachers and peers, all of which contribute to a more favourable learning environment and to their overall academic and personal development (Taylor et al., 2017; Wentzel, 2010). For these reasons, promoting socio-emotional well-being is particularly relevant in inclusive educational contexts, where students with SENs often face additional social, emotional, and relational challenges.

Learning barriers intertwined with discriminatory attitudes in the environment can generate perceptions of lower competence, weakening the personal confidence and sense of belonging of students with SENs. The seriousness of this lies in the importance of self-esteem for the comprehensive development of students, as it influences both their socio-emotional well-being and their academic performance. Positive self-esteem strengthens personal confidence, resilience, and the ability to face challenges, while low self-esteem is often associated with higher levels of anxiety, difficulties in interpersonal relationships, and lower motivation to learn (Marchant et al., 2016). In this sense, promoting self-esteem in the school context not only favours students’ socio-emotional well-being but also enhances their learning and participation in school life. This evidence highlights the need to promote interventions that strengthen self-esteem in students with SENs, recognising its central role in socio-emotional well-being and learning (González-Gil et al., 2020).

In this context, physical activity emerges as a valuable educational and therapeutic resource, as multiple studies have demonstrated its benefits in terms of the perception of competence, self-image, and personal confidence in children and young people with SENs (Piek et al., 2006; Shields et al., 2012). For example, it has been observed that adapted exercise programmes improve the perception of motor skills in students with intellectual and motor disabilities, which has a positive impact on their self-concept and the quality of their social interactions (Bouffard et al., 1996). Similarly, school-based physical education interventions have been shown to improve gross motor skills and perceived motor competence in primary school students, reinforcing the potential of structured physical activity programs to enhance students’ confidence and self-perceptions (Nicolosi et al., 2024). Likewise, reviews highlight that participation in inclusive physical activities strengthens resilience and socio-emotional well-being, while reducing feelings of isolation or exclusion (Groff et al., 2009). Considering the above, integrating physical activity proposals into the school environment can not only promote the physical development of students with SEN, but could also be a key strategy for promoting positive self-esteem and greater socio-emotional well-being. Moreover, research on motivational processes in physical activity shows that students’ perceptions of competence are strongly influenced by the achievement goals promoted in learning environments. Specifically, mastery-oriented goals—focused on personal improvement, effort, and learning—are associated with higher perceived physical competence, greater enjoyment, and more adaptive emotional experiences during physical tasks (Nicolosi et al., 2021).

The school environment is one of the most effective contexts for promoting physical activity, given its massive reach and potential to influence the socio-emotional well-being of schoolchildren (Aguilar-Ozejo & Mujica-Bermúdez, 2024; Martinez-Lopez et al., 2021). In addition to their accessibility, schools have teachers and resources that facilitate the implementation of structured physical interventions (Langford et al., 2015). The benefits of physical activity at school have also been demonstrated in review studies, such as that conducted by Colella et al. (2020) which, through a systematic review, highlights that integrating physical activity into the school day is key to the success of comprehensive health promotion programmes, further improving students’ educational experiences by increasing their self-efficacy and behaviour in the classroom. Evidence indicates that school environments that incorporate physical activity foster quality interpersonal relationships and positive perceptions of competence (Vazou et al., 2017). According to Self-Determination Theory, participation in meaningful physical activities can satisfy the psychological needs for competence, autonomy, and relatedness, promoting self-esteem and socio-emotional well-being (Deci & Ryan, 2000; Ryan & Deci, 2000). Thus, recent literature reviews indicate that physical activity-based interventions have positive effects on psychological well-being and self-esteem in schoolchildren and adolescents, highlighting their potential for inclusive education settings (Li & Huang, 2025; White et al., 2024).

Among the emerging strategies for incorporating physical activity into the school environment, active breaks (ABs), which correspond to brief periods of physical activity during classes, stand out as an effective and efficient strategy for increasing daily physical activity levels without reducing learning time (Kjellenberg et al., 2024). In addition, they contribute to improved concentration, organisation, and working memory, and have even been linked to improvements in academic performance, making them attractive tools for educators (Kjellenberg et al., 2024; Rodríguez-Maimón, 2023; Watson et al., 2017).

However, evidence on the effects of ABs on schoolchildren with SENs is still limited. Preliminary studies suggest that ABs in the classroom can increase physical activity and reduce sedentary behaviour in children with intellectual disabilities, with potential benefits for working memory, but questions remain about their impact on other areas of development, such as motor and socio-emotional skills (Mazzoli et al., 2021), including the effect that ABs could have on the self-esteem and socio-emotional well-being of students with SENs.

Elucidating the effect of ABs on the self-esteem of students with SENs in Chile is relevant due to the specific challenges the country faces in terms of inclusion. In Chile, the identification and educational care of students with SENs is institutionally coordinated through the School Integration Programme (SIP), an inclusive strategy that operates under the principle of providing specialised support within mainstream education establishments (Supreme Decree No. 170/2009) and promotes the equitable participation of students with SENs, in line with international standards of inclusion (Fierro-Saldaña, 2024; MINEDUC, 2010a, 2010b). However, challenges remain in ensuring the socio-emotional well-being of students with SENs in public educational settings. For example, Acuña et al. (2016) analysed the relationship between school coexistence and self-esteem in students with SENs in a school implementing the School Integration Programme, showing that experiences of exclusion or differences in treatment negatively affect their self-concept and sense of belonging. Complementarily, Carvajal Rojas et al. (2019) examined self-esteem in schoolchildren diagnosed with ADHD in the city of Chillán, finding lower levels in girls, which highlights the vulnerability of this population to emotional and gender factors. Therefore, innovative interventions such as Active Classes Chile, which implement active breaks guided by web platforms with curricular content, offer a unique opportunity to combine physical activity, educational inclusion, and socio-emotional development (Robles et al., 2023; Sánchez-Oliva et al., 2022; Zapata-Lamana et al., 2024).

In this context, the present study seeks to examine the effects of a web-based active break programme on school self-esteem among primary school students with SEN in the Biobío region of Chile, and on socio-emotional well-being in the subgroup of first–second-grade students. Based on the prior literature, we hypothesised that participation in active breaks would be associated with improvements in (1) self-esteem and (2) socio-emotional well-being. We anticipated that changes in self-esteem might be more readily detectable in the short term than changes in broader socio-emotional well-being.

## 2. Materials and Methods

### 2.1. Study Design

This study used an experimental design. Specifically, it reports a pre-specified sub-analysis described in the study protocol, focusing on students with SENs who were included in a randomised, controlled, multicentre trial registered at ClinicalTrials.gov (identifier NCT06423404). This study is part of the Active Classes Chile project, which was conducted between March 2022 and October 2024. The study followed the CONSORT guidelines for clinical trials (Butcher et al., 2022) which report on social and psychological interventions (Montgomery et al., 2018) and the SPIRIT guidelines for protocol studies (Chan et al., 2013). The study protocol has been previously published (Zapata-Lamana et al., 2024). This study was reported in accordance with the CONSORT 2025 guidelines for randomised controlled trials and the CONSORT-SPI extension for social and psychological interventions. A completed CONSORT checklist is provided as Supplementary Materials.

Although the parent randomised controlled trial was adequately powered to detect effects of the intervention in the general schoolchild population, it was not specifically designed to detect effects within the subgroup of students with special educational needs (SENs). Randomisation was performed at the school and classroom level and was not stratified by SEN status. Consequently, the present analysis is a pre-specified sub-analysis focused on schoolchildren with SENs and has exploratory objectives.

### 2.2. Participants

**Figure 1:**
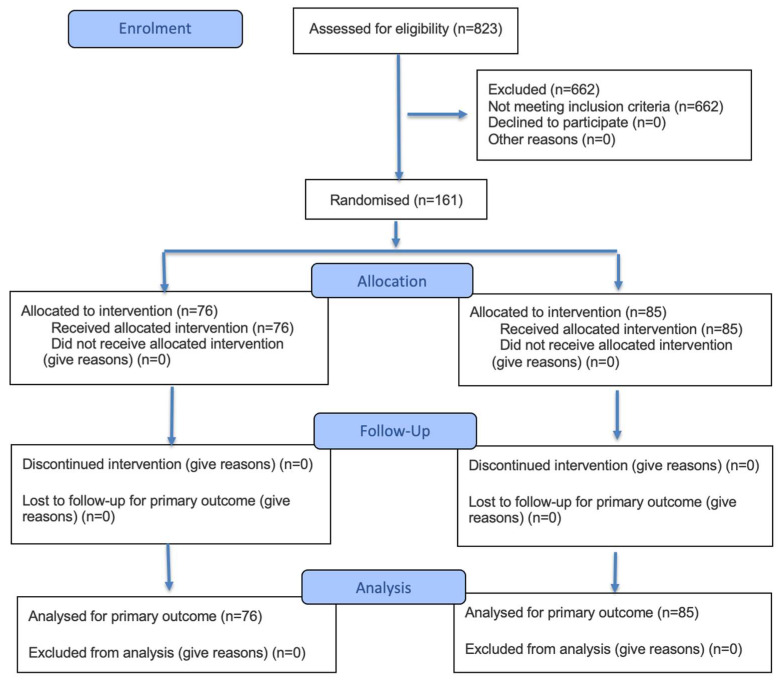
CONSORT flow diagram of participant progress through the randomised controlled trial.

Within the five schools participating in the larger study, students with SENs enrolled in the School Integration Programme (SIP)^1^ were included in this nested study. To this end, children aged 6 to 10 years, from first through fourth grade, were invited to participate in the project. A population of 1080 students with and without SENs from public schools in the Biobío region of Chile was estimated. The sample consisted of 161 students with various special educational needs, including specific learning difficulties, attention deficit disorder, borderline intellectual functioning, language disorders, students on the autism spectrum, intellectual disability, and hearing loss (see Figure 1).

Students with SEN comprised diverse temporary and permanent needs. For the present sub-analysis, SEN participants were analysed as a single group to preserve statistical power, as the study was not designed to support reliable subgroup comparisons by diagnostic category. The sample consisted of primary school students (grades 1 to 4), divided into a control group (85 students) and an experimental group (76 students).

Although this study reports a pre-specified sub-analysis focusing on students with SEN, it is important to note that the parent randomised controlled trial was not originally designed or powered to detect effects specifically within this subgroup. Randomisation was conducted at the classroom level without stratification by SEN status, as the primary aim of the trial was to evaluate the intervention’s effects in the general student population. Furthermore, the SEN group included students with diverse profiles, encompassing both temporary and permanent educational needs (e.g., ADHD, ASD, language disorders, and intellectual disability). This heterogeneity reflects the composition of real inclusive classrooms; however, students were analysed as a single group to preserve statistical power, as the study was not designed to support reliable subgroup comparisons by diagnostic category.

### 2.3. Intervention: Active Classes Platform

The AB programme was designed and developed based on a systematic review previously conducted by the research team (Robles et al., 2023). Based on the conclusions of this review, the optimal type and duration of the intervention, the frequency and intensity of the most effective exercises, and the video-guided modalities with curricular content for the established active breaks were determined.

An interactive web platform was developed for ABs (https://clasesactivas.cl/ (accessed on 1 December 2025). The platform was designed and developed using modern technologies to ensure usability, maintainability, and scalability. The platform has three access profiles: the administrator profile (research team); the school climate coordinator profile (by institution); and finally, the teacher profile (responsible for designing and implementing active breaks in the classroom).

The platform offers a bank of exercises used in ABs, consisting of coordination exercises (bilateral body coordination) and basic motor skills (balance, jumping, movement), previously used (Sánchez-Oliva et al., 2022). These exercises are performed on the platform by two animated characters. Their aim is to encourage identification and adherence among participating students. Both animated characters were developed for this project, and the exercises are performed in pairs to encourage cooperative work and inclusive participation.

The Active Classes platform incorporated a gender perspective, recognising that boys and girls have the same learning potential and the same opportunities for technological development. In addition, the platform hosts an educational resource containing exercises adapted for schoolchildren with reduced mobility (https://clasesactivas.cl/exercises (accessed on 1 December 2025)). Thus, the Active Classes platform has an initial bank of ABs, but each classroom teacher also has a personalised user account that allows them to apply and design their own ABs.

The Active Classes programme was designed for pupils in grades 1 to 4. These breaks last between 5 and 10 min, are of moderate to vigorous intensity, take place twice a day, every day of the school week, and last for 12 weeks. The distribution of ABs was determined taking into account variations in school and class schedules. These should not be at the beginning or end of class, but rather as an interruption within the curricular activity in mathematics, language, social sciences, natural sciences, or English. The active break consists of three phases or moments: ‘welcome and preparation’, ‘core content’ and ‘closure and cool-down’. The core content is supported by exercises adapted for students with low mobility. Although teachers were instructed to implement two active breaks per day, classroom-level fidelity (e.g., frequency and consistency of delivery) was not systematically monitored or recorded. Therefore, variability in adherence and delivery across classrooms may have influenced the magnitude of the observed effects. Future studies should incorporate fidelity monitoring (e.g., teacher logs and/or platform usage analytics) to examine how implementation consistency relates to outcomes.

### 2.4. Variables and Instruments

*(a) School Self-Esteem:* The School Self-Esteem Test (SSET) (Marchant et al., 2016) was used, an instrument developed and validated in Chile by Marchant et al. (2016). The SSET assesses self-esteem in the school context using two versions adapted according to cognitive development level: SSET-Teacher (SSET-T) for students in the 1st and 2nd years of primary school, and SSET-Student (SSET-S) for students in the 3rd and 4th years of primary school.

- *SSET-Teacher (SSET-T):* This version of teacher-rated measures school self-esteem through teacher perception, based on systematic observation of behaviours indicative of self-esteem in the school context. The SSET-T consists of 19 items (e.g., “Feels capable, useful, important to peers”; “Expresses confidence in abilities”) with a 4-point Likert response format: *Rarely* (1 point), *Sometimes* (2 points), *Usually* (3 points), and *Always* (4 points). The total score ranges from 19 to 76 points, with higher scores indicating greater school self-esteem. The instrument has adequate psychometric properties in the Chilean population, with internal consistency of α = 0.94 and test–retest reliability of r = 0.82 (Marchant et al., 2016). Concurrent validity studies with the Coopersmith Self-Esteem Inventory have shown significant correlations (r = 0.67, *p* < 0.001), supporting its construct validity (Marchant et al., 2016).
- *SSET-Student (SSET-S):* This self-assessment version was culturally adapted from the Piers–Harris Children’s Self-Concept Scale (Piers, 1984) for the Chilean population. The SSET-S comprises 23 dichotomous items (e.g., “I am bothered by my appearance, how I look”; “I am lucky”) with a Yes/No response format. Each response consistent with positive self-esteem receives 1 point, while responses indicative of low self-esteem score 0. The total score ranges from 0 to 23 points, with higher scores reflecting higher self-esteem. The SSET-S exhibits satisfactory psychometric properties, with internal consistency of α = 0.81 and temporal stability of r = 0.76 over a four-week interval (Marchant et al., 2016). Construct validity has been supported by confirmatory factor analysis, identifying a unidimensional structure of school self-esteem with adequate fit indices (CFI = 0.92, RMSEA = 0.06).
  The differences in self-esteem scores between the different school levels should be interpreted considering the type of informant rather than as an evolutionary change, since the evaluations carried out by teachers and the students’ self-reports capture complementary but different perspectives of the same construct.

*(b) Socio-Emotional Well-being scale:* The Self-Report of Socio-Emotional Well-being for Children from Pre-kindergarten to 2nd Grade (Lira et al., 2005) was used, an instrument developed and validated in Chile to assess socio-emotional well-being in early education through self-report. The instrument assesses four dimensions: adaptation to school work, social adaptation, self-esteem, and perception of competence. It uses a visual response format with pictorial scales (smiley faces/emoticons) that facilitate understanding in children with limited reading skills and is administered through a structured interview in which the evaluator reads each item while the student indicates their response.

The study implemented a differentiated assessment strategy according to educational level. To measure school self-esteem (n = 161): (a) 1st- and 2nd-grade students (n = 64) were assessed using SSET-T, answered by head teachers based on systematic observation of the student, given the metacognitive limitations of this age group for reliable self-reporting; (b) 3rd- and 4th-grade students (n = 97) were assessed using the self-reported SSET-S, taking advantage of the emerging introspective abilities at this age. Socio-emotional well-being was assessed exclusively in 1st- and 2nd-grade students (n = 64) using an assisted self-report, given that the psychometric properties of the instrument are validated only for this age range.

Two assessment approaches were used for self-esteem to ensure developmental appropriateness: teacher ratings in grades 1–2 (SSET-T) and student self-reports in grades 3–4 (SSET-S). Accordingly, differences in self-esteem scores across grade levels should be interpreted primarily in light of informant source rather than developmental change, as teacher ratings and student self-reports capture complementary but distinct perspectives on the same construct (Denham et al., 2007; Harter, 2012; Marsh & Craven, 2006).

### 2.5. Covariates

The covariates analysed in this study were determined using self-report questionnaires used in the Active Classes Chile project, which have been used previously (Camargo et al., 2015). Sociodemographic variables were included, such as gender (boy or girl), age, area of residence (urban, rural), distance from the school (1 to 5 blocks, 6 to 10 blocks, 11 to 15 blocks, and more than 15 blocks), access to a park/sports field/gym near the home (no access, with access); school variables such as school hours (morning, afternoon, full day) and school uniform (tracksuit, traditional, mixed); and family variables such as education (none, primary, secondary, and higher).

### 2.6. Statistical Analysis

Baseline characteristics of participants were described using means (M) and standard deviations (SD). Data normality was checked using the Shapiro–Wilk test. For self-esteem, a mixed repeated-measures ANOVA was conducted, including Time (pre vs post), Group (experimental vs control), and Reporter Type (teacher-rated vs self-reported) as factors, as well as their interactions. For socio-emotional well-being, which was only assessed via student self-report in 1st–2nd grade, a two-way repeated-measures ANOVA was performed considering Time and Group as factors. A significance level of *p* < 0.05 was set for all analyses. Effect sizes were interpreted based on partial eta squared (η^2^_p_). Statistical analyses were carried out using JASP (version 0.19.3).

## 3. Results

The main sociodemographic characteristics of the students who participated in the study are reported below. A total of 161 schoolchildren (7.8 ± 1.1 years, 32.3% girls) with special educational needs from schools in the Biobío region of Chile participated in the intervention (experimental group n = 76, 47.3%; control group n = 85, 52.7%). Most of the students in both groups lived in urban areas (88.1%) and did not participate in extracurricular workshops (72.1%) (see Table 1).

Before examining the effects of the intervention, descriptive statistics for the variables evaluated are presented according to group and measurement time. Table 2 shows the means and standard deviations corresponding to self-esteem scores—reported by teachers in the 1st and 2nd years and through self-reporting in the 3rd and 4th years—as well as socio-emotional well-being for students in the 1st and 2nd years. In general terms, self-esteem scores tend to be higher in the subsequent measurement in most groups, while emotional well-being remains relatively stable.

### 3.1. Results on Self-Esteem

**Figure 2:**
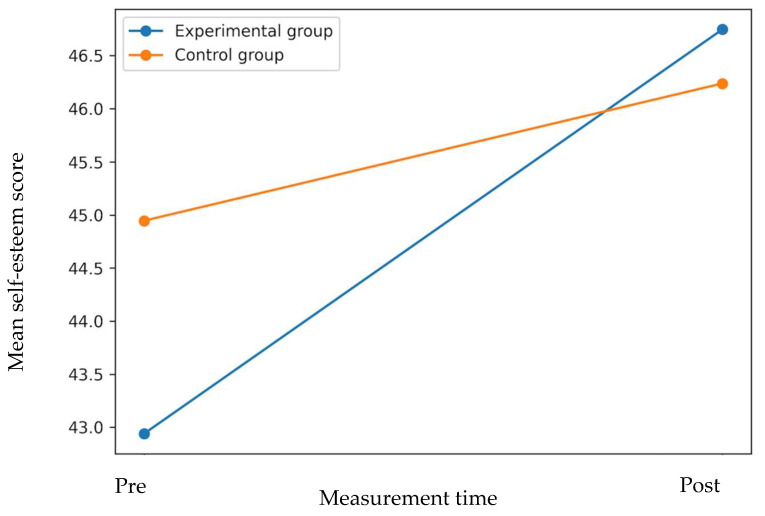
Changes in self-esteem from pre- to post-intervention in experimental and control groups. Mean self-esteem scores increased over time in both groups, with a greater improvement observed in the experimental group following the active break intervention.

The three-factor repeated-measures analysis (Time × Group × Reporter Type) showed a significant main effect of Time, F_(1, 157)_ = 22.72, *p* < 0.001, η^2^_p_ = 0.126, indicating that Time accounted for 12.6% of the variance of self-esteem levels. A significant interaction between Time × Group was also observed, F_(1, 157)_ = 5.43, *p* = 0.021, η^2^_p_ = 0.033, indicating a small Time × Group effect on self-esteem in the experimental group compared to the control group. Likewise, the Time × Reporter Type interaction was significant, F_(1, 157)_ = 10.96, *p* = 0.001, η^2^_p_ = 0.065, indicating that the magnitude of change differed depending on who reported self-esteem. Specifically, the increase was more evident in grades where the report was made by teachers (first–second) than in those where it was self-reported by students (third–fourth). In contrast, the Time × Group × Reporter Type interaction did not reach statistical significance, F_(1, 157)_ = 2.22, *p* = 0.139, indicating that although the patterns of change were different between teacher reports and student self-reports, these differences did not vary sufficiently between the experimental and control groups within each reporting source. With regard to the effects between subjects, Group (Experimental versus control) did not show significant differences in overall self-esteem levels (*p* = 0.452), while Reporter Type did, F_(1, 157)_ = 39.19, *p* < 0.001, η^2^_p_ = 0.200, showing that the scores reported by teachers were systematically higher than those self-reported by students. Therefore, differences observed between grade levels should be interpreted primarily as reflecting differences in reporting sources rather than age-related or developmental differences in self-esteem (see Figure 2).

### 3.2. Results in Socio-Emotional Well-Being

In the case of socio-emotional well-being, assessed through self-report in first- and second-year primary school students, no significant differences were observed over time, F_(1, 62)_ = 0.334, *p* = 0.468, indicating no relevant changes between the initial and final measurements. The Time × Group interaction also did not reach statistical significance, F_(1, 62)_ = 2.905, *p* = 0.093. However, the main effect of Group was significant, F_(1, 62)_ = 4.883, *p* = 0.031, indicating differences between the experimental and control groups in overall levels of socio-emotional well-being, regardless of the time of assessment. Taken together, these results show that, unlike what was observed in self-esteem, students did not perceive substantive changes in their emotional or social experience during the period evaluated, which appears to remain stable in the short term (see Table 3).

## 4. Discussion

This study reports a pre-specified sub-analysis of a multicentre randomised controlled trial evaluating a web-based active break programme in primary school students with SEN in the Biobío region of Chile. Outcomes included school self-esteem (Grades 1–4) and socio-emotional well-being (Grades 1–2).

The main findings show a statistically significant increase in self-esteem levels after the intervention, with a main effect of time indicating overall improvements in this dimension regardless of group and type of report (teacher report or student self-report). In addition, the Time × Group interaction showed that the increase was greater in students in the experimental group, while the Time × Reporter Type interaction showed that the improvements were more pronounced when self-esteem was reported by teachers (in first and second grade) than when it was self-reported by students (third and fourth grade). In contrast, socio-emotional well-being did not show statistically significant changes in first- and second-grade students, suggesting that, unlike teachers’ perceptions of self-esteem, the students themselves did not report relevant changes in their emotional or social experience during the evaluation period. These results reinforce the idea that self-esteem may be more sensitive to active participation and positive feedback interventions, while improvements in emotional well-being may require more prolonged processes, systematic relational support, and specific emotional regulation strategies.

The findings are consistent with previous evidence highlighting the positive impact of physical activity and guided movement on children’s self-perception and socio-emotional adjustment in educational and actual contexts (Kjellenberg et al., 2024; Watson et al., 2017). The improvement in self-esteem observed, especially when assessed by teachers, may be related to visible changes in commitment, motor self-efficacy, and willingness to participate in school activities, all aspects that teachers can identify more clearly in daily interaction (Turda, 2021). Likewise, from the perspective of achievement emotions (Pekrun, 2006) it is possible that structured physical–motor activities generated more opportunities for students to experience success, recognition and enjoyment, thus strengthening positive evaluations of their abilities. On the other hand, the increase in self-esteem is consistent with Self-Determination Theory (Deci & Ryan, 2000; Ryan & Deci, 2000) according to which the satisfaction of the needs for competence, autonomy and relatedness promotes psychological well-being. In this way, by incorporating adapted activities, cooperative work and elements of teacher personalisation, the *Active Classes Chile* platform probably facilitated experiences of success that reinforced students’ self-worth (Sánchez-Oliva et al., 2022).

However, it should be noted that, although the effects on self-esteem were statistically significant, the effect sizes observed were small, indicating that the intervention explains a small proportion of the variance in this variable. This is consistent with the literature, which indicates that changes in psychosocial constructs in students with SENs tend to be gradual and sensitive to multiple contextual factors (Vazou et al., 2017). In this sense, the physical activity videos may have generated specific positive experiences that contributed to an observable improvement by teachers, but were probably not sufficient to modify the overall self-esteem of the students in a more comprehensive and sustained manner. Likewise, it may be that the small effect size is due to the intervention having an effect on a more specific area of self-esteem, such as physical self-concept, or it may be influenced by the brevity of the intervention, differences in reporting sources, and the presence of diverse educational needs that require individualised support. Self-esteem represents a construct of self-evaluation that may be more sensitive to structured experiences of success, while socio-emotional well-being encompasses broader processes of emotional regulation and social functioning, which tend to require interventions of longer duration and with a more marked relational emphasis. Therefore, the absence of short-term changes should not be interpreted as evidence of ineffectiveness of the intervention, but rather as a manifestation of the complexity inherent in these constructs. Therefore, although the findings are encouraging, more extensive, contextualised, and multifaceted interventions are needed to achieve greater impacts on the self-esteem of this population.

Although the observed effect sizes for self-esteem were small (e.g., η^2^p = 0.033 for the Time × Group interaction), small-to-moderate effects are common in school-based programmes targeting psychosocial outcomes (Durlak et al., 2011; Taylor et al., 2017). In inclusive classroom settings, even modest improvements in self-esteem—particularly those observed in routine teacher–student interactions—may represent meaningful incremental gains in engagement and perceived competence. Accordingly, the present findings should be interpreted as modest but potentially educationally relevant benefits achieved through a low-cost, scalable classroom strategy.

Regarding socio-emotional well-being, the absence of a significant Time × Group effect suggests that the intervention did not produce detectable changes in students’ self-reported socio-emotional well-being over the study period. At the same time, the significant main effect of Group indicates overall level differences between experimental and control groups across measurement occasions, which may reflect baseline differences or unmeasured contextual factors rather than an intervention-related change. Two non-exclusive interpretations are therefore plausible. First, active breaks may have limited effectiveness for improving socio-emotional well-being among students with SENs under the current implementation conditions. Second, potential effects may not have been captured, as changes in broader socio-emotional domains may require greater intensity, specificity, and duration.

As previously noted, findings from Mazzoli et al. (2021) suggest that the benefits of ABs for students with intellectual disabilities remain preliminary and appear to be context-dependent. Moreover, the absence of improvements in the experimental group may reflect moderating factors such as classroom climate, teacher expectations, or heterogeneity within SENs (TNEEs vs. PSENs). This reinforces the need for studies that examine not only the feasibility of ABs, but also the characteristics they must have to be more effective in real-world inclusive settings (Zapata-Lamana et al., 2024).

This study has several limitations that should be considered when interpreting the findings. First, because the parent randomised controlled trial was not powered to detect effects within the SEN subgroup, the present analyses should be interpreted as exploratory, and statistical power was limited. This limitation is particularly relevant for socio-emotional well-being, which was assessed only in grades 1–2 (n = 64), further reducing power to detect small-to-moderate effects and potentially contributing to null findings. Second, the generalizability of the findings is restricted to students aged 6 to 10 with SENs attending public schools in two regions of Chile, which limits extrapolation to other educational levels, sociocultural contexts, or types of establishments. Third, the heterogeneity of SEN profiles—including temporary and permanent needs (e.g., ADHD, ASD, language disorders, and intellectual disability)—may have influenced responsiveness to the intervention and attenuated observable effects.

For analytic purposes, students with SENs were examined as a single group to preserve statistical power, as the study was not designed to support reliable subgroup comparisons by diagnostic category. Fourth, self-esteem was assessed using different informants across grades (teacher report in grades 1–2 vs student self-report in grades 3–4), meaning that results reflect different perspectives on the construct and should be interpreted cautiously when comparing grade levels. Future research should include longer follow-ups, incorporate stratified designs or SEN-specific adaptations where feasible, and examine whether combining active breaks with explicit socio-emotional supports enhances effectiveness (Taylor et al., 2017; Zapata-Lamana et al., 2024).

(Zapata-Lamana et al., 2024) Despite these limitations, the study extends previous evidence by providing data on active breaks in an underrepresented group—students with SENs—beyond findings from general school populations (Kjellenberg et al., 2024; Watson et al., 2017). Our results also differ from the pilot evidence in children with intellectual disability reported by Mazzoli et al. (2021), suggesting that effects may represent across outcomes and implementation conditions. Finally, the findings support the feasibility of delivering video-guided active breaks via web platforms in real classroom settings (Robles et al., 2023; Sánchez-Oliva et al., 2022) and indicate a modest improvement in self-esteem among students with SEN (Robles et al., 2023).

Thus, video-guided ABs could represent a scalable and low-cost strategy to strengthen the self-esteem of students with SENs, aligned with initiatives such as the School Integration Programme (SIP) (Fierro-Saldaña, 2024; MINEDUC, 2010a). These results provide guidance for teachers and schools seeking to implement active methodologies within inclusive educational frameworks, while also informing policymakers about the realistic scope of such interventions. However, it must be noted that findings on socio-emotional well-being warn that the mere incorporation of ABs does not guarantee broad socio-emotional benefits. Its implementation should be integrated into broader frameworks that include teacher training, classroom climate monitoring, and explicit socio-emotional development activities (Li & Huang, 2025; White et al., 2024).

In line with the theoretical distinction raised in the Introduction, the results obtained reinforce the idea that self-esteem and socio-emotional well-being respond to different dynamics of change. While self-esteem seems to be more sensitive to structured experiences of active participation and positive reinforcement, socio-emotional well-being involves deeper and more relational processes that are difficult to modify in the short term. This distinction suggests that the absence of significant short-term changes in socio-emotional well-being should not be interpreted as intervention failure, but rather as reflecting the gradual and relational nature of this construct.

## 5. Conclusions

This study adds evidence that video-guided active breaks can yield **modest improvements in self-esteem** among primary school students with SENs. By contrast, **no significant change** was observed in socio-emotional well-being; in this sample, overall well-being levels differed between groups regardless of time, suggesting that socio-emotional outcomes may be less sensitive to short-term active-break implementation alone. From a practical perspective, Active Classes Chile appears **feasible and adaptable** for classroom delivery and may serve as a low-cost complementary strategy to support self-esteem in inclusive settings. Future research should examine how implementation conditions (e.g., fidelity and classroom context) and the addition of more explicit socio-emotional or relational components influence broader socio-emotional outcomes.

## Figures and Tables

**Table 1:** Characteristics of schoolchildren.

Variables	Total (n = 161)	Experimental Group (n = 76; 47.3%)	Control Group (n = 85; 52.7%)
Age, M ± SD	7.8 ± 1.1	7.9 ± 1.2	7.6 ± 1.0
Gender, n (%)			
Boys	109 (67.7)	50 (65.7)	59 (69.5)
Girls	52 (32.3)	26 (34.3)	26 (30.5)
Zone, n (%)			
Urban	142 (88.2)	63 (82.8)	79 (92.9)
Rural	19 (11.8)	13 (17.2)	6 (7.1)
School			
1	43 (26.7)	0 (0.0)	43 (50.5)
2	42 (26.2)	0 (0.0)	42 (49.5)
3	20 (12.3)	20 (26.4)	0 (0.0)
4	31 (19.3)	31 (40.8)	0 (0.0)
5	25 (15.5)	25 (32.8)	0 (0.0)
Grade, n (%)			
1	27 (16.8)	13 (17.1)	14 (16.4)
2	37 (23.0)	16 (21.1)	21 (24.7)
3	51 (31.7)	21 (27.6)	30 (35.4)
4	46 (28.5)	26 (34.2)	20 (23.5)
After-school workshop			
Yes	45 (27.9)	24 (31.5)	21 (24.7)
No	116 (72.1)	52 (68.5)	64 (75.2)

**Table 2:** Pretest and posttest descriptive statistics of self-esteem and socio-emotional well-being by group and grade level.

Variable	Grade Level and Reporter	Group	n	Pre M (SD)	Post M (SD)
Self-Esteem	1st–2nd (Teacher-rated)	Experimental	29	40.69 (1.78)	41.76 (1.50)
Self-Esteem	1st–2nd (Teacher-rated)	Control	35	41.34 (3.74)	41.63 (3.38)
Self-Esteem	3rd–4th (Student self-report)	Experimental	46	44.36 (7.12)	49.89 (9.94)
Self-Esteem	3rd–4th (Student self-report)	Control	51	47.42 (8.45)	49.40 (8.87)
Socio-emotional Well-Being	1st–2nd (Student self-report) **	Experimental	29	24.17 (1.63)	23.86 (3.07)
Socio-emotional Well-Being	1st–2nd (Student self-report) **	Control	35	22.40 (3.05)	23.03 (2.29)

Note: Scores reflect standardised scale totals. Higher values indicate greater perceived self-worth or socio-emotional well-being. Group = intervention condition (Experimental vs. Control). ** Socio-emotional well-being was assessed only in 1st–2nd-grade students using assisted self-report, given that the instrument is validated exclusively for this age group. Socio-emotional well-being was only collected for 1st–2nd-grade students.

**Table 3:** Results of repeated-measures ANOVA for self-esteem and socio-emotional well-being by group and reporter type.

Effect	df	*F*	*p*	η^2^p
Self-Esteem (Repeated-Measures ANOVA)				
Time	1, 157	22.71	<0.001	0.126
Time × Group	1, 157	5.43	0.021	0.033
Time × Reporter Type	1, 157	10.95	0.001	0.065
Time × Group × Reporter Type	1, 157	2.21	0.139	0.014
Group (Between Subjects)	1, 157	0.56	0.452	0.004
Reporter Type (Between Subjects)	1, 157	39.19	<0.001	0.200
Socio-Emotional Well-Being (Repeated-Measures ANOVA)				
Time	1, 62	0.33	0.468	0.005
Time × Group	1, 62	2.90	0.093	0.045
Group (Between Subjects)	1, 62	4.88	0.031	0.073

## Data Availability

The data presented in this study are available on request from the corresponding author. The data are not publicly available due to ethical and privacy restrictions.

## Note

1 The SIP is a programme that provides guidelines for achieving inclusion for all students through guidelines in line with global organisations that promote the inclusion of diversity at all levels of the education system (Fierro-Saldaña, 2024). This programme considers the participation of schoolchildren with temporary needs (such as attention deficit disorder, specific learning difficulties, borderline intellectual disability and language disorder) as well as permanent needs (autism spectrum disorder, intellectual disability, sensory and motor disability). The Ministry of Education in Chile defines Transitional Special Educational Needs (TNEEs) as those in which students need extraordinary help and support to access or progress in the curriculum for a certain period of their schooling, while students with Permanent Special Educational Needs (PSENs) are those who experience barriers to learning and participation throughout their schooling as a result of a disability (MINEDUC, 2010a, 2010b).

## Supplementary Materials

The following supporting information can be downloaded at: https://www.mdpi.com/article/10.3390/bs16030459/s1, (Hopewell et al., 2025) CONSORT 2025 checklist of information to include when reporting a randomised trial.
